# Minor hyperthyroidism with normal levels of thyroid-stimulating hormone receptor antibodies: a case report

**DOI:** 10.25122/jml-2023-0458

**Published:** 2024-02

**Authors:** Andrey Valerievich Ushakov

**Affiliations:** 1 Ushakov Thyroid Clinic, Moscow, Russia

**Keywords:** hyperthyroidism, Graves’ disease, thyroid ultrasound, Doppler, autonomic nervous system, TSHR-Ab, PSV-STA

## Abstract

The present report describes for the first time a case of diffuse hyperthyroidism in a 30-year-old female patient who had normal levels of thyroid-stimulating hormone receptor antibodies (TSHR-Ab), slightly elevated plasma levels of thyroid hormones, and slightly increased thyroid blood flow. Seven years before, after severe stress, she had Graves’ disease with elevated plasma levels of TSHR-Ab. The patient’s recent medical history included mental stress and autonomic dysfunction. This report describes a mild form of hyperthyroidism in terms of elevated plasma levels of thyroid hormones and Doppler ultrasonography data; this condition was first defined as ‘minor hyperthyroidism’. The examination data suggest a probable secondary role of the immune system and primary role of the autonomic nervous system in the pathogenesis of Graves’ disease.

## INTRODUCTION

In cases of diffuse (non-nodular) hyperthyroidism, called Graves’ disease (GD), excessive numbers of antibodies against thyroid-stimulating hormone receptors (TSHR-Ab) are often detected in the patients’ serum samples. This finding is considered key in the pathogenesis of GD [1]. In addition, these patients exhibit high plasma levels of free thyroxine (FT4) and triiodothyronine (FT3), as well as high blood flow intensity in the thyroid vasculature, and high peak systolic velocity (PSV) values in the thyroid arteries, as detected using Doppler ultrasonography [2]. These findings not only indicate the key role of the immune system in the onset and development of GD but also suggest that TSHR-Ab influences thyroid blood flow via unknown mechanisms [3,4].

However, some clinical cases of diffuse hyperthyroidism present clinical evidence that do not support the hypothesis that immune mechanisms have a major role in the pathophysiology of the disease, and these require more explanation. These evidence include slightly elevated thyroid hormone levels, normal TSHR-Ab levels, and low intensity thyroid blood flow, which was revealed in our patient.

## CASE PRESENTATION

A 30-year-old female patient (height, 164 cm; weight, 55 kg) presented to our clinic with the chief complaint of sleep disturbances (i.e., difficulty falling asleep) and no history of medications. Her arterial blood pressure at rest in the sitting position was 108/76 mm Hg, and her heart rate was 91 bpm. Blood serum examination conducted on 23 June 2022 revealed the following: thyroid-stimulating hormone (TSH), 0.083 mU/l (normal range, 0.4–4.0); FT4, 19.7 pmol/l (normal range, 9.0–19.0); FT3, 7.2 pmol/l (normal range, 3.0–5.6); total thyroxine (TT4), 137.5 pmol/l (normal range, 62.6–150.8); total triiodothyronine (TT3), 2.2 nmol/l (normal range, 0.9–2.2); TSHR-Ab < 0.21 IU/l (normal values, <1); thyroid peroxidase antibodies (TPOAb) < 3.0 U/ml (normal values, <4.1); antithyroglobulin antibodies (TgAb) < 3.0 U/ml (normal values, <5.6); erythrocytes 4.5 million/µl (normal range, 3.8–5.1); hemoglobin 13.1 g/dl (normal range, 11.7–15.5); normal leukocyte count; erythrocyte sedimentation rate 9 ml/h (normal range, 0–20); and C-reactive protein 0.9 mg/l ( normal values, <5).

**Figure 1:**
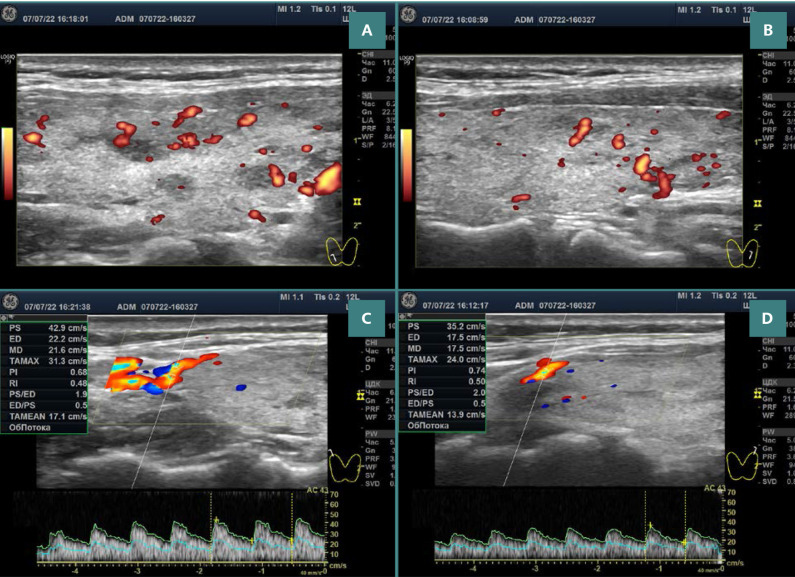
Doppler ultrasonography images of both lobes of the thyroid gland. A,B, Slightly increased blood flow intensity in both lobes (higher intensity is seen in the right lobe). C,D, PSV-STA is 42 cm/s and 35 cm/s in the right and left arteries, respectively (in contrast, the common carotid arteries showed a PSV of 67 cm/s and 42 cm/s in the right and left arteries, respectively, in which the complete arteries were measured at the same level).

The thyroid ultrasound revealed a slight increase in gland volume (20.6 ml [11.^5^ + 9.^1^]) with a significant mass of isoechogenic tissue (up to 95%), slight swelling of the stroma (very low hypoechogenicity), and a few lymphoid lobules (several pseudonodes). The ultrasound Doppler revealed a slightly increased blood flow intensity and PSV in the superior thyroid arteries (Figure 1).

The closest relatives were questioned, but no cases of thyroid disease were found. According to the medical history of the patient, GD was experienced for the first time in 2010. After 1 year of taking thiamazole (Thyrozol) with a gradual dose reduction, the patient’s hormonal metabolism normalized, and until 2015, euthyroidism was maintained with good health condition (without medication). In 2015, GD exhibited a pronounced relapse because of substantial mental stress caused by the death of a beloved person. Concurrently, an increase in TSHR-Ab levels was noticed. Nevertheless, the situation improved gradually, and by the end of 2017, the hormonal status of the patient returned to euthyroidism. The remission continued until the beginning of 2022, when a single episode of mental stress caused a disease attack that was accompanied by palpitations, dull pain in the chest, shortness of breath, tremor in the limbs, and sweating. After a few hours, the attack resolved completely. Subsequently, the patient’s condition improved; however, insomnia persisted.

At the primary examination conducted on 4 April 2022 — for in vitro fertilization procedure, the serum analysis unexpectedly revealed subclinical hyperthyroidism with normal TSHR-Ab. There were no laboratory signs of thyroiditis (autoimmune or subacute), the only manifestations were insomnia. The patient reported that she did not take any drugs in the past few years. In the last year there have been no infectious diseases or colds.

## DISCUSSION

There are relatively few reports in the literature on cases of hyperthyroidism with normal TSHR-Ab levels. However, according to previous studies, these patients account for 5–7% of all patients with diffuse hyperthyroidism [5–8]. Most researchers have linked these cases to a non-autoimmune variant of the inherited disease associated with genetic alterations [9,10]. Meanwhile, some specialists consider this course of hyperthyroidism as a variant of GD caused by non-immunological and non-genetic mechanisms because elevated TSHR-Ab levels are found subsequently in these patients [5,11]. Therefore, in such patients, other clinical manifestations should be investigated to identify the essence of diffuse hyperthyroidism when TSHR-Ab levels are not elevated.

The co-occurrence of slightly elevated thyroid hormones and slight thyroid hypervascularization is the key feature of this case. This suggests that the thyroid gland was mildly overstimulated. However, the origin of such stimulation needs to be identified. Notably, the antithyroid antibody levels were within the normal range, and the TSH level was almost zero.

Diffuse hyperthyroidism is most likely caused by autonomic nervous system (ANS) activation, which can be triggered under psychological stress conditions and can affect the thyroid vasculature, immune response, and hormone production [12–15]. The level of ANS excitation may vary from low to medium and high. Therefore, this case demonstrates the possibility of ANS stimulation by the central nervous system, followed by thyroid overstimulation. Hence, the small elevation in ANS excitation affects the body and thyroid accordingly, causing minor stimulation of thyrocyte activity and thyroid vasculature when the level is low.

As suggested by this report, such cases of mild thyroid overload may not require immune system activation. Moreover, the effect of ANS on the immune system may not be strong enough. This may be the reason why the level of TSHR-Ab involved in thyroid regulation also remains low. Simultaneously, under conditions of low-level ANS stimulation, the thyroid parenchyma is also slightly depleted, with no increase in TPOAb and TgAb levels.

It is possible to hypothesize that a considerable increase in ANS excitation will result in increased thyroid stimulation and a highly pronounced hyperthyroidism. The appearance of elevated TSHR-Ab serum levels (along with an increase in FT4 and FT3 levels) in patients with diffuse hyperthyroidism with normal TSHR-Ab levels suggests that the immune system has a secondary role in the etiology of GD. Moreover, this conclusion is supported by the evidence of gradual decrease and normalization of TSHR-Ab levels along with those of FT4 and FT3 during GD remission.

The present case cannot be attributed to a hereditary variant of the disease owing to the lack of a family history of thyroid diseases and the fact that in 2015, an increase in TSHR-Ab levels associated with pronounced hyperthyroidism was observed. This aspect represents the strength of the study; however, a limitation of the study is the lack of genetic research.

Thus, the involvement of the ANS in the pathogenesis of GD enables the logical integration of numerous clinical evidence revealed in this and other cases into a single coherent pathogenesis. It allows for example, to understand cases of unilateral hyperthyroidism, which can be explained by the unilateral excitation of the peripheral ANS. Notably, ANS controls the innervation of each lobe’s conduction system separately, therefore this process cannot be explained by metabolic pathogenesis alone [16].

## CONCLUSION

A slight increase in FT3 and FT4 levels in hyperthyroidism is appropriately referred to as ‘minor hyperthyroidism’. It is very likely that the ANS has a crucial role in the pathogenesis of GD, and the immune regulation is only additionally involved in the disease. Further studies involving all clinical evidence are required to clarify the described model of pathogenesis of this disease.
